# Molecular cytogenetic differentiation of paralogs of Hox paralogs in duplicated and re-diploidized genome of the North American paddlefish (*Polyodon spathula*)

**DOI:** 10.1186/s12863-017-0484-8

**Published:** 2017-03-02

**Authors:** Radka Symonová, Miloš Havelka, Chris T. Amemiya, William Mike Howell, Tereza Kořínková, Martin Flajšhans, David Gela, Petr Ráb

**Affiliations:** 1 Laboratory of Fish Genetics, Institute of Animal Physiology and Genetics, Czech Academy of Sciences, 277 21, Liběchov, Czech Republic; 2 Research Institute for Limnology, University of Innsbruck, Mondseestr. 9, Mondsee, Austria; 3 University of South Bohemia in České Budějovice, Faculty of Fisheries and Protection of Waters, South Bohemian Research Center of Aquaculture and Biodiversity of Hydrocenoses, 389 25 Vodňany, Czech Republic; 4 Benaroya Research Institute & University of Washington, Seattle, WA 98101 USA; 5 Department of Biological and Environmental Sciences, Samford University, 800 Lakeshore Drive, Birmingham, AL 35229 USA

**Keywords:** HoxA/D paralogs mapping, Sturgeon whole genome duplication, Ancient fish genome, Rediploidization

## Abstract

**Background:**

Acipenseriformes is a basal lineage of ray-finned fishes and comprise 27 extant species of sturgeons and paddlefishes. They are characterized by several specific genomic features as broad ploidy variation, high chromosome numbers, presence of numerous microchromosomes and propensity to interspecific hybridization. The presumed palaeotetraploidy of the American paddlefish was recently validated by molecular phylogeny and Hox genes analyses. A whole genome duplication in the paddlefish lineage was estimated at approximately 42 Mya and was found to be independent from several genome duplications evidenced in its sister lineage, i.e. sturgeons. We tested the ploidy status of available chromosomal markers after the expected rediploidization. Further we tested, whether paralogs of Hox gene clusters originated from this paddlefish specific genome duplication are cytogenetically distinguishable.

**Results:**

We found that both paralogs HoxA alpha and beta were distinguishable without any overlapping of the hybridization signal - each on one pair of large metacentric chromosomes. Of the HoxD, only the beta paralog was unequivocally identified, whereas the alpha paralog did not work and yielded only an inconclusive diffuse signal. Chromosomal markers on three diverse ploidy levels reflecting different stages of rediploidization were identified: quadruplets retaining their ancestral tetraploid condition, semi-quadruplets still reflecting the ancestral tetraploidy with clear signs of advanced rediploidization, doublets were diploidized with ancestral tetraploidy already blurred. Also some of the available microsatellite data exhibited diploid allelic band patterns at their loci whereas another locus showed more than two alleles.

**Conclusions:**

Our exhaustive staining of paddlefish chromosomes combined with cytogenetic mapping of ribosomal genes and Hox paralogs and with microsatellite data, brings a closer look at results of the process of rediploidization in the course of paddlefish genome evolution. We show a partial rediploidization represented by a complex mosaic structure comparable with segmental paleotetraploidy revealed in sturgeons (Acipenseridae). Sturgeons and paddlefishes with their high propensity for whole genome duplication thus offer suitable animal model systems to further explore evolutionary processes that were shaping the early evolution of all vertebrates.

**Electronic supplementary material:**

The online version of this article (doi:10.1186/s12863-017-0484-8) contains supplementary material, which is available to authorized users.

## Background

The North American (*Polyodon spathula*) and Chinese paddlefish (*Psephurus gladius*), i.e. fishes with paddle-like snout, are the only extant representatives of an early radiation of ray-finned fishes recognized as the family Polyodontidae within the order Acipenseriformes [1]. This ancient lineage, i.e. sturgeons, shovelnoses (Acipenseridae) and paddlefishes, represents an archaic group known to be at least as old as the early Jurassic (some 200–175 Mya). Polyodontids and acipenserids diverged from one-another in the Jurassic period ~180–140 Mya [2]. Paddlefishes form a distinct monophyletic clade within Acipenseriformes as evidenced by molecular phylogeny [3].

There is a consistent body of evidence on the chromosome number of the North American paddlefish but discordant data on its genome size, yet all authors consider this species palaeotetraploid. Namely, Dingerkus and Howell [4] based on karyotyping of 31 cells from two paddlefish males found the 2n = 120 and reported a karyotype apparently composed of twelve quadruplets of macro- and 72 microchromosomes and thus hypothesized a tetraploid origin in this species. Later, the 2n = 120 was reconfirmed and nuclear DNA content corresponding to genomes of sturgeons with 2n = 120 was reported by Zhang et al. [5]. Birstein et al. [6] re-examined nuclear DNA content of this species and confirmed its palaeotetraploid status. The Chinese paddlefish (*Psephurus gladius*) has 2n = 120 and nuclear DNA content corresponding to sturgeon genomes with 2n = 120 was reported [5]. All available data indicate a palaeotetraploid origin of the paddlefish lineage, similarly as in a number of sturgeon species [7].

The cytogenetic analyses of sturgeons and paddlefishes is challenging since, in addition to macrochromosomes, extremely small-sized microchromosomes typically constitute a substantial proportion of the acipenseriform karyotype and their morphology has long been characterized as indistinguishable [8]. However, a recent work [9] reports morphology of these small-sized chromosomes that would have been called “microchromosomes”. Further details on sturgeon genetics and cytogenetics related to ploidy level were summarized by Havelka et al. [10] and Trifonov et al. [11].

Sturgeons generally exhibit a remarkable propensity for hybridization and polyploidization resulting in viable and even fertile highly polyploid individuals and inter-specific (allopolyploid) hybrids [12–15]. Each such whole genome duplication (WGD) is followed by a rediploidization [16]. During this process, a partial retention of 4n features is observable, like chromosome numbers and their external morphology (e.g. [17]). Distinct signs of ongoing and to different extent advanced rediploidization can be found in other cytogenomic markers – e.g. HoxA/D sequences in paddlefish [12] and microsatellite studies in sturgeon [18, 19].

Recently, molecular analyses of paralogs of both HoxA and HoxD gene clusters demonstrated a specific WGD event in the paddlefish lineage dated about 42 million years ago (Mya) (for more details on dating [12]) and independent from the multiple WGDs in sturgeons [19]. Other comparable events among ray-finned fish are the salmonid specific WGD (called SR) dated about 88 Mya [20] and the teleost specific WGD (TSGD) dated about 320 Mya [21]. Paddlefish thus offers a model system complementary to others to study consequences of WGD by molecular cytogenetics in the light of clear indications of secondary rediploidization at the molecular level in HoxA/D genes clusters (e.g. [12]).

Hox genes clusters are expressed along the anteroposterior axis of all bilaterians and play a key role in animal development [22]. In vertebrates, four paralogous Hox clusters (Hox A, B, C, and D) originate from a single Hox cluster of the last common ancestor of vertebrates and cephalochordates ([23], [24] reviewed in [25]). These four paralogs arose by two ancestral consecutive WGD events known as R1 and R2 that occurred in the vertebrate stem lineage around 525 Mya [24, 26]. The paddlefish specific WGD gave rise to a further order of paralogs called here α and β, respectively, as introduced by [12].

In this study, using FISH (fluorescence in situ hybridization) we co-hybridized the HoxAα with HoxAβ and HoxDα with HoxDβ of these paddlefish specific paralogs (BAC DNA produced by Crow et al. [12]) to paddlefish chromosomes. This was combined with FISH with ribosomal (rRNA) genes, with conventional chromosome banding and microsatellite analysis to explore consequences of the paddlefish specific WGD and the subsequent rediploidization. The available markers and analyses, including those based on external chromosomal morphology, enabled us to assess the tetraploidy retention versus the extent of rediploidization on the level of coding regions, repetitive sequences, heterochromatin accumulations supplemented with microsatellite analysis.

## Results

### Karyotype and chromosome analysis

**Fig. 1:**
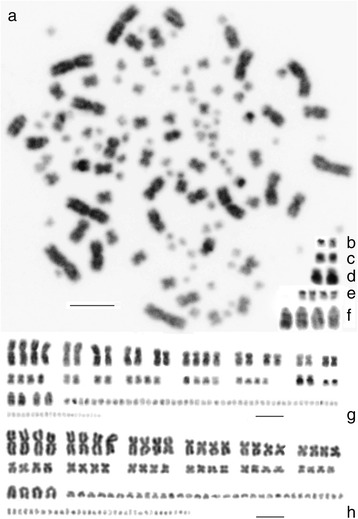
Summary of karyological data. DAPI stained metaphase reversed to *black* and *white* (**a**), Chromomycin A_3_ positive small submetacentric chromosomes, reversed to *black* and *white* (**b**), DAPI positive small submetacentric chromosomes (**c**), DAPI positive middle-sized subtelocentric chromosomes (**d**), AgNOR positive chromosomes (**e**), acipenseriform marker chromosomes - the largest acrocentric chromosomes, the first two of them with two distinct DAPI positive bands (**f**). Karyotype of the DAPI stained metaphase with macrochromosomes arranged into quadruplets, semi-quadruplet and duplets based on the DAPI signal (**g**). Karyotype of Giemsa stained chromosomes with macrochromosomes arranged only into quadruplets (**h**). Scale bars equal 10 μm

Karyotype analysis re-confirmed the diploid chromosome number 2n = 120 and enabled an arrangement of chromosomes into quadruplets, doublets and semi-quadruplets where possible (Fig. 1). This arrangement reflected the external morphological features of chromosomes and also information retrievable from the DAPI (i.e., AT-rich regions)/CMA_3_ (Chromomycin A3, GC-rich regions specific) fluorescent staining. Firstly, a CMA_3_^+^ arm of a pair of small sub metacentric macrochromosomes (Fig. 1b). Secondly, DAPI^+^ arm of a small submetacentric chromosomes (Fig. 1c). Thirdly, an interstitial DAPI^+^ band on the larger arm of a pair of submetacentric chromosomes (Fig. 1d). DAPI staining demonstrated that there is no other pair of submetacentric chromosomes with comparable external morphology and banding pattern. Further, two pairs of the largest “acipenseriform” acrocentric chromosome markers were distinguishable based on their morphology in the tetraploid condition. However, the DAPI fluorescence revealed AT-rich signals accumulated in form of a double-band in only two of these four chromosomes (Fig. 1f). The AgNO_3_ staining (visualizing chromosomes with the active ribosomal genes, the AgNORs) consistently yielded a quadruplet of transcriptionally active major ribosomal sites on four small metacentric macrochromosomes (Fig. 1e). The two CMA^3+^ signals (Fig. 1b) did not co-localize with the active NOR sites (Fig. 1e).

### FISH experiments

**Fig. 2:**
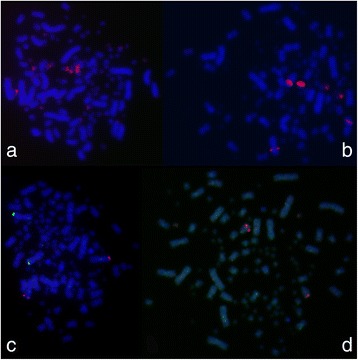
FISH with rDNAs and Hox clusters. FISH with 28S rDNA (**a**, *red*), 5S rDNA (**b**, *red*) and FISH with BACs of the paralogs Hox-A alpha (**c**, *red*) and Hox-A beta (**c**, *green*) and of the paralogs Hox-D beta (**d**, *red*) and Hox-D alpha (**d**, *green* – the diffuse character of this signal causes a greenish coloration of almost all chromosomes). Scale bars equal 10 μm

The FISH with 28S ribosomal DNA (rDNA) showed up to 12 signals on mostly small-sized macrochromosomes or microchromosomes (Fig. 2a). The FISH with 5S rDNA revealed signals on three morphologically different pairs of chromosomes (Fig. 2b): i) small dot-like signals interstitially on one arm of a pair of large metacentric chromosomes; ii) large signals on a whole arm of a pair of small metacentric macrochromosomes, and iii) dot-like signals on a pair of small metacentric macrochromosomes. FISH with telomeric repeats (TTAGGG) n did not reveal any interstitial telomeric sites (not shown).

FISH with HoxAα/β and HoxDβ gene clusters, respectively, showed a clear 2n pattern of the respective paralogs (Fig. 2c-d). Namely, the HoxA α paralog was physically mapped to the telomeric region of a pair of large metacentric chromosomes (Fig. 2c, red signals), the HoxA β paralog mapped also to telomeric region of another pair of large metacentric chromosomes (Fig. 2c, green signals) with no overlapping signals. The HoxD β paralog mapped to the telomeric region of one pair of the largest acrocentric chromosomes (Fig. 2d, red signals). The HoxD α paralog yielded an inconclusive interspersed FISH signal and it was impossible to localize this region even with using competitor DNA (Fig. 2d, green and diffuse greenish signals on majority of chromosomes).

### Integration of karyological and molecular cytogenetic markers

Integrating all analyzed markers, the following groups of chromosomes (labeled with abbreviations for later reference) were identified to compose the karyotype: M1 quadruplet of the largest metacentric chromosomes of equal size (according to external morphology); M2 semi-quadruplet of slightly smaller large metacentric chromosomes based on HoxA paralogs mapping; M3 semi-quadruplet of meta- to submetacentric chromosomes of intermediate size including one smaller and one larger doublet respectively based on size and 5S rDNA FISH signals; SM1 semi-quadruplet of submetacentric chromosomes of intermediate size; SM2 semi-quadruplet; SM3 semi-quadruplet; SM4-5 (semi-quadruplets/doublets of small meta- to submetacentric chromosomes, these chromosomes are difficult to classify into separate groups and pair with the exception of the AgNOR quadruplet; ST1 semi-quadruplet/doublet of a conspicuous pair of submeta- to subtelocentric chromosomes with a remarkable DAPI^+^ band on the larger arm. This pair of chromosomes lacks any counterpart in its size and morphological category, however, it can be linked with a pair of small metacentric chromosomes with a distinct DAPI band on one arm; A1 semi-quadruplet of medium-size acrocentric chromosomes (the acipenseriform acrocentric markers). There is a DAPI^+^ double-band on two of these chromosomes and the HoxD β paralog FISH signal on the other two chromosomes; m1 doublet of very small chromosomes (microchromosomes) with a clear CMA_3_^+^ band; m2 doublet of very small chromosomes (microchromosomes) with a clear DAPI^+^ band. In this way, we identified 54 macrochromosomes (or microchromosomes with features enabling their identification) and 66 microchromosomes (i. e. small chromosomes without any cytogenetic features).

Contrary to these observations yielded by fluorescent stainings, AgNOR staining and C-banding, the Giemsa staining produced only rough and uniform external chromosomal morphology corresponding approximately to tetraploid condition. There were no apparent characteristics enabling any possibility to distinguish the aforementioned quadruplets, semi-quadruplets and duplets (Fig. 1h).

**Fig. 3:**
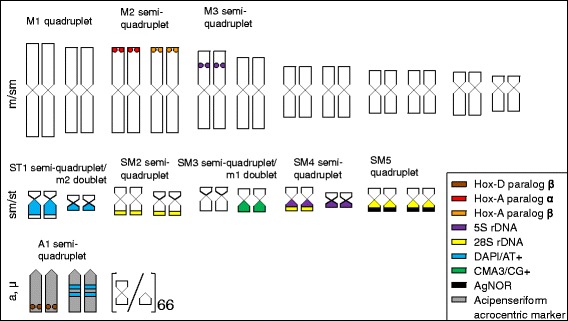
Ideogram summarizing all chromosomal markers investigated in this study

All cytogenetic data are visualized and summarized in the Fig. 3.

To sum up, the ancestrally palaeotetraploid paddlefish chromosomal complement (2n = 120) represents a complex mosaic structure consisting of characters with 1/retained tetraploidy (AgNORs); 2/partially retained tetraploidy, i.e. apparent ancestral tetraploidy with clear signs of secondary diploidization (acipenseriform acrocentric marker, HoxA and HoxD loci); 3/diploidized characters (DAPI^+^ and CMA_3_^+^ heterochromatic sites); and 4/characters with apparent chromosomal re-arrangements (5S rDNA sites). The situation in 5S rDNA sites did not match neither to ancestral tetraploidy nor to the diploidized condition.

### Quantification of repetitive sequences in BAC clones used as FISH probes

Proportion of repeats in the BAC DNA used as FISH probes can be potentially crucial for functionality of FISH experiments since repeats interspersed throughout genome can yield signals unspecific for the BAC DNA under study. In Table 1, we summarize repeats proportion for each BAC DNA.

**Table 1:** Overview of BACs of Hox paralogs used as FISH probes

Clone ID *sensu* [12]	BAC	Paralogs	GenBank Accession Nr.	Size (bp)	Repeats (%)
BAC231C24	1816	Hox-D 8-13α	JX280946.1	22.134	5.57
BAC249G23	1817	Hox-D 8-13β	JX280945.1	33.595	9.17
BAC352P4	1818	Hox-A 1-13α	JX448769.1	131.867	10.66
BAC370N10	1819	Hox-A 1-13β	JX448770.1	139.159	10.48

### Microsatellite genotyping

**Fig. 4:**
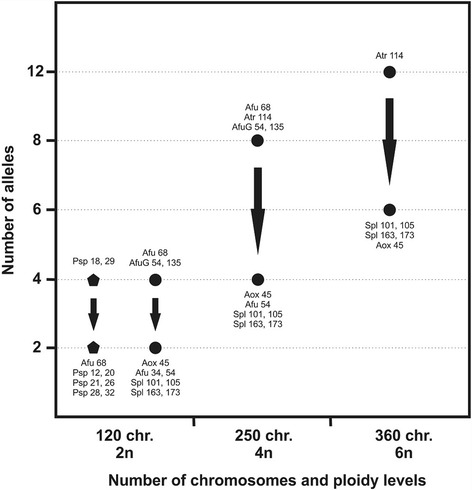
Microsatellite analysis of ploidy level. Microsatellite data (number of alleles) in *Polyodon spathula* and comparison with sturgeons related to chromosomes numbers and ploidy levels provide evidence of partial genome rediploidization in *P. spathula* () and sturgeons (●). The figure is based on data from this study and previous studies [18, 28]

From all together eleven tested sturgeon microsatellite markers, only the locus Afu 68 [27] displayed consistent amplification in *P. spathula*. This locus exhibited diploid allelic band pattern across all analyzed samples. Similarly, *P. spathula* specific loci (see Additional file 1, Supplementary Methods) had diploid allelic band patterns. An exception to this general pattern was shown at the locus Psp-29 [28] for which more than two alleles were observed (Fig. 4; Additional file 2, Supplementary Results).

## Discussion

This study demonstrated an in situ localization of three of four available paralogs of the HoxA and HoxD gene clusters in *P. spathula*. Both HoxA paralogs were clearly distinguishable without any FISH signal overlaps showing that the sequence divergence was high enough to enable their unequivocal discrimination under high stringency conditions. The sequence divergence between the full coding regions varied among gene clusters ranging from 2.12% (Hox-A11) to 10.94% (Hox-D13) [12]. The proportion of repetitive sequences was identified to be approximately 10% where ascertainable (Table 1). The repetitive sequences, if specific for the Hox clusters, might have contributed to discrimination of Hox paralogs. On the other hand, the failure in localization of the HoxD α can also be ascribed to accumulation of repetitive sequences that were spread also throughout the rest of genome and thus prevented from an accurate localizing of this paralog. This result contributes as a comparative reference to the future attempts to localize paralogous regions and presents an example of linking genomic approach with molecular cytogenetics. Further, our results made it possible to better assess genome evolution after the paddlefish specific WGD on the finer-scale level in combination with other cytogenetic markers and to compare it with the outer chromosomal morphology based on conventional cytogenetics as shown by [4].

Ray-finned fishes provide an outstanding model to investigate WGDs and their consequences because of their complex evolutionary history involving among vertebrates unprecedented propensity for hybridization and polyploidization and their genome plasticity tolerating high variability in genome size and chromosome number [29].

Two basic ways of chromosomal evolution following a WGD event were documented among ray-finned fishes: 1/conserving of the chromosome numbers after WGD which exists in more than 50% of teleosts after the TSGD [30], in polyploid cyprinid lineages [31] and in most acipenseriforms [19], and 2/extensive chromosomal re-arrangements leading to diversified chromosome numbers, e.g. in salmonids (reviewed by [32]). Other lineages known to have experienced WGD [33] need to be cytogenetically documented.

**Fig. 5:**
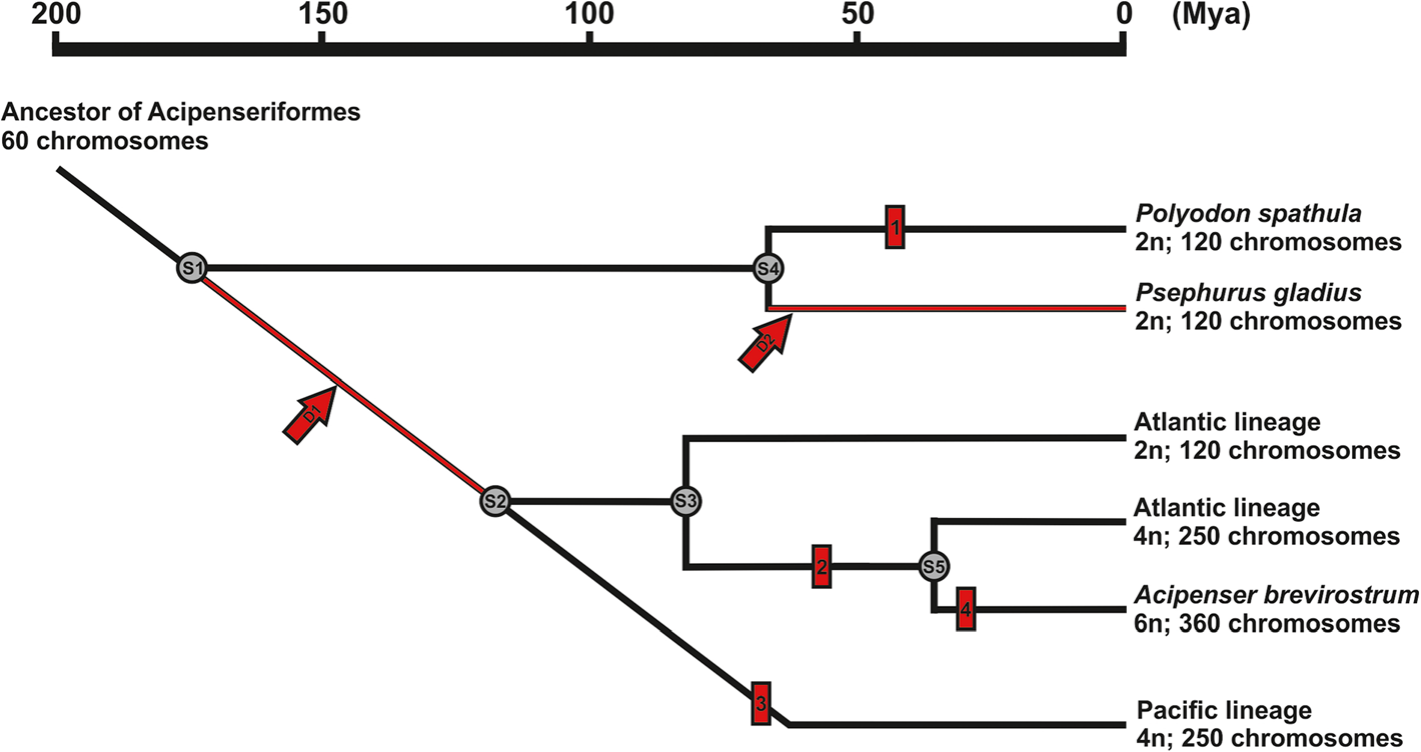
Suggested main events in evolution of Acipenseriformes. S1 = split of Polyodontidae and Acipenseridae (~170 Mya); S2 = Split of Atlantic and Pacific lineage in Acipenseridae (~121 Mya); S3 = split of 2n and 4n species within Atlantic lineage (~80 Mya); S4 = Split of *Polyodon spathula* and *Psephurus gladius* (~68Mya); S5 = Split of *Acipenser brevirostrum* (~36 Mya). Whole genome duplication (WGD) events are: 1 = WGD specific to *Polyodon spathula* (60 → 120 chromosomes;~ 42 Mya [12]); 2 = WGD in Atlantic lineage (120 → 250 chromosomes; ~ 53 Mya); 3 = WGD in Pacific lineage (120 → 250 chromosomes; ~ 70 Mya); 4 = WGD specific to *Acipenser brevirostrum* (250 → 360 chromosomes ~ 35 Mya); D1 = the first WGD in Acipenseridae (60 → 120 chromosomes) had to take place between ~ 170 Mya and ~ 121 Mya; D2 = probable WGD (60 → 120 chromosomes) specific to *Psephurus gladius*. The data are based on study by Peng et al. [2] and Crow et al. [12]

Acipenserids, the sister lineage of polyodontids, underwent at least three rounds of lineage specific WGDs [19, 34, 35]. The first one occurred in their common (already extinct) ancestor with 2n = 60 leading to a ~120-chromosomes lineage with presently unknown dating. The second lineage specific WGD took place separately in the Atlantic sturgeon lineage (~53 Mya) and in the Pacific lineage (~70 Mya). The third WGD is unique to the Shortnose sturgeon (*Acipenser brevirostrum*) dated ~35 Mya [2]. *Polyodon spathula * inhabits the same range as some species of the Atlantic sturgeon lineage. The *P. spathula* specific WGD is supposed to have occurred ~42 Mya [12] which is a timing somewhat similar to the WGD of the Atlantic sturgeon lineage [2]. Acipenserids split from polyodontids ~ 170 Mya and the divergence time between the Pacific and the Atlantic lineage appears as about 121 Mya [2]. Assuming WGD specific to *P. spathula* [12] and no presence of WGD specific to Atlantic lineage species with 120 chromosomes [2], the first specific WGD in Acipenseridae had to take place between split of polyodontids from acipenserids (~170 Mya) and split of Atlantic and Pacific lineage of Acipenseridae (~121 Mya; Fig. 5).

Chromosome numbers and their outer morphology in both acipenserids and polyodontids remain mostly conserved in the post-WGD situation [4]. Regarding the longevity estimated in the paddlefish WGD, this conservatism might be explained by two possible factors and/or by combination thereof: 1/significantly reduced rate of molecular evolution (both mitochondrial and nuclear) that was reported in both coding and non-coding sequences as well as in chromosomal evolution [36, 37]. It can be assumed that also the rate of secondary diploidization in paddlefish and acipenserids might have been influenced by this generally reduced rate of evolution; 2/morphology of chromosomes undergoing WGD should be taken into account. Once exclusively or mostly metacentric chromosomes undergo a WGD (as in acipenseriforms and also in cyprinids), there is a narrower spectrum of potential chromosome rearrangements to be employed within the duplicated sets of chromosomes. This factor might have contributed to the situation shown here on paddlefish chromosomes. Whereas e.g. in salmonids, chromosomes of the hypothetical 2n ancestor might have been composed of mostly acrocentric chromosomes [38] which facilitates chromosomal evolution via Robertsonian fusions. Such mechanism was recently described in human acrocentrics harboring rDNA - under certain circumstances acrocentrics physically link via their centromeres and become fusogenic [39]. From our previous studies we know that salmonids possess amplified to extremely amplified rDNA sites mostly in centromeres of acrocentric chromosomes [40, 41]. These might have been the factors contributing to the numerous centric fusions described in all salmonid lineages [32] and at the same time contributing to the conservatism preserving the post-WGD chromosome number in the paddlefish. However, more detailed analyses on the finer scale as shown here by molecular cytogenetic approach or by sequencing of Hox genes paralogs [12] revealed advanced stages of diploidization in coding (HoxA/D gene clusters) and some non-coding regions (accumulation of AT^+^- and GC^+^-rich heterochromatic regions; microsatellites). This indicates dynamic processes on multiple levels in the acipenseriform lineage despite the chromosomal morphology prevented from Robertsonian fusions. On the other hand, as evidenced by the persisting of tetraploid state in AgNORs and four active nucleoli per nucleolus, it is advantageous to retain the elevated structural and functional ploidy level in rRNA genes. The multiplied number of non-active 28S rDNA sites on mostly small chromosomes suggests tendencies to spread these regions across chromosomes as evidence in other fishes (e.g. [41, 42]). Our findings of a complex mosaic of diploid, tetraploid and intermediary chromosomal features are in line with the concept of segmental paleotetraploidy revealed recently in the sterlet (*Acipenser ruthenus*) by chromosome painting [9].

In paddlefish, the re-diploidization process on the karyotype level might have occurred without a change of chromosome counts but by finer-scale re-arrangements of some chromosomes. The fact that some chromosomes and markers still retain the quadruplet nature (i.e. ancestral 4n = 120), supports the hypothesis that the diploid ancestor before the WGD event possessed 2n = 60 as proposed for the whole group of Acipenseriformes [4, 7].

For further comparisons of consequences of WGDs, more recent entirely polyploid lineages and families within Cypriniformes [33] as e.g. *Cyprinus carpio* (about 12 Mya; David et al. 2003) are available among ray-finned fish. This would be one of the most recent genome duplications among vertebrates indicating a higher incidence of WGD in fishes than in other vertebrate groups [43]. These authors report partially duplicated genome structures and disomic inheritance despite clearly tetraploid chromosome number (2n = 100) in *C. carpio* demonstrating the complexity of genome evolution in this group. There are also reports on recurrent allopolyploidization events within the *Carassius* complex [44] proving thus the suitability of ray-finned fish that provide almost a continuum in WGD events on the time scale. However, there are no comparable results available yet to assess the process of rediploidization.

### Origin of the acipenseriform acrocentric chromosome marker and its relation to the ploidy level

One of the four Hox clusters - the HoxD β paralog - mapped to two of the four largest acrocentric chromosomes while both of the HoxAα/β paralogs to four large metacentric chromosomes (the M2 group). Therefore we assume that the largest acrocentric chromosomes originated from one of the large metacentric chromosomes that also represent the ancestral location of Hox clusters. These acipenseriform acrocentric marker chromosomes are known to reflect the ploidy level and used to ploidy level estimation (*sensu *[13]). They occur in all sturgeons (details on the online database, http://sveb.unife.it/it/ricerca-1/laboratori/geneweb). Presumably, the Hox clusters were residing on one pair of chromosomes before the 1R, i.e. the first vertebrate WGD (as shown by [45] in *Amphioxus*) and on two pairs before the 2R (the second vertebrate WGD). Hence, there were four pairs of Hox bearing chromosomes before the paddlefish specific WGD, which resulted in eight pairs (i.e. HoxA-D/α-β; here, we have investigated four of them and localized three of them). The largest acrocentric chromosomes might thus have arisen by fission of one pair of the metacentric chromosomes. This is in line with the assumption that the numerous metacentric chromosomes represent the ancestral chromosomal morphology. The few acrocentric chromosomes may represent rather exceptional derivatives of metacentrics of more recent origin. This scenario is supported by the fact that no interstitial telomeric sites were found in paddlefish and therefore nothing suggests the opposite way of origin of metacentric chromosomes by centric fusions as proposed by Birstein [46]. This scenario finds its support in *Acipenser ruthenus*, where a single acrocentric pair shares FISH signals of a painting probe derived from the seventh pair of the large metacentrics [9]. This suggests that the fission of the ancestral pair of metacentric chromosomes might have happened already before the split of polyodontids and acipenserids or repeatedly in both lineages. These authors explicitly deny the presence of the second pair of the largest acrocentrics in individuals they investigated [9]. On the other hand, the other two largest acrocentric chromosome are clearly observable in *A. ruthenus* as published by other authors (see the above mentioned online database maintained by F. Fontana). Hence, there are apparent lineage specific trends and differences in the chromosomal evolution between the polyodontids and acipenserids.

### Microsatellite analyses are in line with cytogenetic findings

Microsatellite data in Acipenseriformes were recently presented by Havelka et al. [18]. They identified sturgeon species in palaeotetraploid condition that were functionally diploid showing a diploid allelic band pattern in some microsatellite loci whereas residual tetraploid pattern in other ones. They further identified functionally tetraploid palaeooctaploids showing tetraploid patterns in some loci and residual octaploid patterns in other ones. Finally, a special situation was described in the functionally hexaploid palaeododecaploid *A. brevirostrum* which also showed hexaploid vs. residual dodecaploid patterns (for details [18]). Such observation of coexistence of diploid and tetraploid or tetraploid and octaploid allelic band patterns in one genome of sturgeon species might reflect functional rediploidization as an ongoing process in this fish lineage (e.g. [19]). This process is expected in polyploids until their complete rediploidization [16]. However, even in fully diploidized genomes, residual evidence for polyploid ancestry (e.g. residual polysomy) is occasionally observed (e.g. in salmonids, [47]). Since paleotetraploid acipenseriform species were considered to be basal group of Acipenseriformes [3], the process of rediploidization probably reaches further than in paleooctaploid species [19]. In light of all these facts, the observation of the duplicated locus Psp-29 in this study and coexistence of diploid and tetraploid allelic band patterns reported by Heist et al. [28] at several microsatellite loci of *P. spathula* supported our observation of partial rediploidization in *P. spathula* genome from the molecular point of view.

The estimation of locus ploidy by microsatellite genotyping may suffer from inbreeding of analyzed individuals. This might be the case of our microsatellite data as all analyzed individuals originated from a pet shop and we could not exclude their relatedness. Except the locus *Psp 18*, the estimated ploidy of analyzed loci was in accordance with Heist et al. [28] providing confidence for our conclusion based on microsatellite data. Heist et al. [28] suggested tetraploidy for the locus *Psp 18*, while we did not observe more than two alleles at the locus (Additional file 2, Supplementary Results). This inconstancy may be caused by close relatedness of analyzed individuals in our study.

Our study presented here and the recently published work [9] performed in sterlet (*Acipenser ruthenus*) represent the first steps towards a better understanding of processes involved in the rediploidization after WGD events in Acipenseriformes. Both works, although utilizing slightly different approaches of molecular cytogenetics, intersect in presenting a complex mosaic structure consisting of 2n and 4n chromosomes segments referred to as “segmental paleotetraploidy” by [9]. Both works thus show the complexity and at the same time the importance of this issue.

## Conclusions

We have shown that the paddlefish *Polyodon spathula*, in which the chromosome numbers and chromosome morphology remain mostly conserved in the post-WGD situation, do nevertheless show signs of ongoing re-diploidization. By combining karyological and molecular cytogenetic markers we were able to distinguish three diverse ploidy levels: tetraploidy (AgNORs), partially retained tetraploidy with secondary diploidization (acipenseriform acrocentric marker, HoxA and HoxD loci) and diploidized characters (DAPI^+^ and CMA_3_^+^ heterochromatic sites). Accordingly, the altogether 120 chromosomes can be arranged into quadruplets, semi-quadruplets and doublets. We suggest that paddlefishes are similar to their relatives, sturgeons, in their propensity for genome duplication and subsequent rediploidization, and that both groups have a good prospect as vertebrate models for further exploration of these processes.

## Methods

### Material and metaphase chromosome preparation

Eight individuals of *P. spathula* of unknown sex examined cytogenetically in this study are summarized in the Table 2. They were sacrificed by overdose of anaesthetic 0.5% Phenoxyethanol (v/v, SIGMA), blood was taken for leucocytes cultivation and fin clips for microsatellite genotyping. Only a fraction of samples yielded metaphase spreads of sufficient quality to be used in molecular cytogenetic analyses.

**Table 2:** List of Polyodon spathula specimens analysed cytogenetically

Indivuals processed for chromosomes	Origin of specimens	Individuals used for molecular cytogenetics (# metaphases)
1–7/2008	University of South Bohemia in Ceske Budejovice	1 (15)/2008
1–6/2009	University of South Bohemia in Ceske Budejovice	1 (5), 5 (10)/2009
1–8/2012	Pet shop in Mlada Boleslav, originally from Hungary	1 (2), 4 (2), 5 (14)/2012
1–8/2013	Pet shop in Mlada Boleslav, originally from Hungary	7 (11), 8 (13)/2013

All fish examined in this study, chromosome preparations, and DNA and tissue samples are deposited in the Laboratory of Fish Genetics, Institute of Animal Physiology and Genetics, Czech Academy of Science (LFG, IAPG, CAS), Liběchov as voucher specimens and reference samples.

Additionally, fin clips were taken from twenty four individuals originating from a pet shop in 2014 and these were subsequently processed for microsatellite genotyping.

The leucocytes were cultured and chromosome spreads prepared according to the protocol [48] with some modifications described in [13]. To increase the chance of obtaining usable chromosome preparations, some individuals were processed using a direct method according to [13].

### Chromosome staining and karyotype analysis

Paddlefish chromosomes were analysed by Giemsa staining and specific staining and banding methods combined with FISH mapping. Namely, buffered Giemsa (pH 7.0, 5%, 5 min) was performed to assess chromosome quality and provide comparison with earlier chromosomal studies. Subsequently, chromosomes were destained by incubation in fixative for 3 min at RT and briefly washed by distilled water. After air-drying, CMA_3_ and DAPI fluorescent staining was performed sensu Sola et al. [49]. Finally, the AgNO_3_ staining sensu Howell and Black [50] and C-banding sensu Haaf and Schmid [51] were performed. Separately DAPI- and CMA_3_-stained chromosomes were converted into black and white images, inverted and arranged into karyotypes.

### Fluorescence in situ hybridization (FISH) and signal detection

Whole genomic DNA (gDNA) was isolated from blood, using DNeasy Blood & Tissue Kit (Qiagen, Hilden, Germany) according to the manufacturer’s instructions. The 28S rDNA and 5S rDNA were PCR amplified from gDNA according to [52] and [53]. The amplified fragments were purified using either the QIAquick PCR purification kit (Qiagen) or Qiagen Gel Extraction Kit, according to the manufacturer’s instructions. Aliquots of the purified 28S rDNA were used in a sequencing reaction using BigDye® Terminator v1.1 Cycle Sequencing Kit (Life Technologies) and subsequently analysed at ABI Prism® 3700 Genetic Analyzer. Additionally, the same PCR products were sequenced by the Macrogen Inc. (Seoul, South Korea). Since a preliminary sequence screening has shown an intra-individual sequence variation of the 5SrDNA, purified products from three independent PCR runs were subjected to cloning using the Qiagen PCR Cloning Kit. The procedure followed the manufacturer’s instructions, except for using a half of the recommended amount of the vector and competent cell: 20 ng of the PCR product was ligated to 25 ng of the pDrive cloning vector and a 1 μl aliquot of the obtained ligation-reaction mixture was used to transform 25 μl of Qiagen EZ Competent Cells. These were cultured for 12–24 h at 37 °C on LB agar plates containing Ampicillin (100 μg/ml). Randomly selected colonies from each transformed cell lineage were then transferred into the liquid LB medium and cultivated overnight on a shaking platform at 37 °C. Plasmids were isolated and 5SrDNA was extracted using the QIAprep Spin Miniprep Kit (Qiagen). The extract DNA was sequenced in the same way as previously described for 28S rDNA.

The nucleotide sequence data have been deposited in GenBank (under accession numbers KM103731-KM103735: KM103731 18S rDNA, KM103732 28S rDNA, KM103733- KM103735 5S rDNA).

DNA probes were indirectly labelled with biotin-16-dUTP and digoxigenin-11-dUTP (both Roche Diagnostics, Mannheim, Germany) through labelling PCR re-amplification of the previously sequenced PCR products. Reactions were performed in 50 μl total volume containing 1× reaction buffer, 2 mM MgCl_2_, labelled dNTP nucleotide mix (dATP, dCTP, dGTP each 12.5 μM, dTTP 8.5 μM, dUTP conjugated with a hapten - biotin-16-dUTP or digoxygenin-11-dUTP (both Roche), final concentration 4 μM), forward and reverse primer (0.4 μM each), 1.25 U of Taq polymerase (all reagents from Top-Bio, Prague, Czech Republic) and approximately 100 ng of PCR product as template DNA. The dNTP nucleotide mix was prepared as a premix containing 5 μl dATP, dCTP, dGTP (each 2.5 nM) and 3.4 μl dTTP (2.5 nM), 4 μl dUTP (1 mM) conjugated with a hapten, 27.6 μl PCR water in total volume 50 μl.

FISH with telomeres were performed using the Star*FISH Concentrated Human Chromosome Pan-Telomeric Painting Probe directly labelled with the Cy3 fluorescent dye (Cambio, Cambridge, UK) according to manufacturer’s instruction.

The BAC clones of paralogous gene clusters for each of the HoxA and HoxD (Table 1, including accession numbers in GenBank) were produced by Crow et al. (2012) [12] and provided for this study. The BAC DNA was labelled indirectly (biotin-16-dUTP and digoxigenin-11-dUTP) by nick translation using the Nick Translation Mix (Roche) according to the manufacturer’s instructions. The precipitation and resuspension of the probe as well as aging (3–12 h at 37 °C and 30 min at 65 °C) and pepsin treatment of the chromosome preparations, hybridization and detection were performed as described in [54]. FISH with the BAC DNA of both paralogs of HoxA and HoxD were performed with and without competitor DNA derived from the Siberian sturgeon (*Acipenser baerii*) genomic DNA in excess 20–50 times of the probed BAC DNA.

The nomenclature of respective HoxA/D α and β paralogs was adopted from [12]. This nomenclature reflects the fact that these paralogs are products of the paddlefish specific WGD event.

### Quantification of repetitive sequences in used BAC clones

DNA sequences of BAC clones used as FISH probes originating from [12] (Table 1) have been retrieved from the online “Nucleotide” NCBI database https://www.ncbi.nlm.nih.gov/nuccore according to their GenBank Accession Numbers (Table 1). These sequences have been subjected to screening for interspersed repeats and low complexity DNA sequences with the online tool RepeatMasker [55] using *Danio rerio* as the DNA source for sequence comparison, ‘abblast’ search engine, and otherwise default settings. Produced reports are summarized in this study (Table 1) and all complete reports are archived by RS.

### Microscopy and image processing

Chromosome preparations were observed with the AX70 Olympus microscope equipped with a standard fluorescence filter set and captured with a black and white CCD camera separately for each fluorescent dye. Digital images were then pseudo-coloured (blue for DAPI, red for Rhodamine or Cy3, green for Fluorescein or FITC and CMA_3_) and processed in Adobe Photoshop, version CS5. Karyotypes were produced using the IKAROS software (Metasystems, Germany).

### Microsatellite analyses

Twenty four individuals were processed for microsatellite genotyping. The genomic DNA was extracted from fin clips stored in 96% molecular grade ethanol by the NucleoSpin®tissue kit (Macherey-Nagel, Germany) following manufacturer protocol. Nineteen microsatellite markers were tested for amplification using standard gradient – PCR. These markers are listed in Additional file 1 (Supplementary methods).

Markers, which consistently amplified, were selected for subsequent analyses. To avoid fluorescent labeling of each forward primer, forward primers within each of the primer sets possessed a 5′ prime end tail (M13R). During PCR, a fluorescently labelled primer (M13R) was added to the standard amplification reaction [56]. Detailed PCR protocol is listed in Additional file 1 (Supplementary methods). The level of genome reduplication/reduction was investigated as described by [18].

## Additional files

Additional file 1: Supplementary Methods. Microsatellite markers tested for amplification in the present study and PCR protocol. (DOC 45 kb)
Additional file 2: Supplementary Results. Results of microsatellite genotyping, comparison of number of alleles, allele frequencies by locus. (PDF 83 kb)

## Abbreviations

BAC (DNA): Bacterial artificial chromosome DNA
CMA3: Chromomycin A3 stain
Cy3: Cytochrome 3
DAPI: 4′, 6-diamidino-2-phenylindole stain
FISH: Fluorescence in situ hybridization
FITC: Fluorescein isothiocyanate
gDNA: Genomic DNA
HoxA/D: Homeobox gene clusters A/D
Mya: Millions of years ago
PCR: Polymerase chain reaction
rDNA: Ribosomal deoxyribonucleic acid
WGD: Whole genome duplication
