# Supplementary material for: Molecular cytogenetic differentiation of paralogs of Hox paralogs in duplicated and re-diploidized genome of the North American paddlefish (Polyodon spathula)
Source: BMC Genet. 2017 Mar 2;18:19. doi: 10.1186/s12863-017-0484-8 (PMC5335500; doi:10.1186/s12863-017-0484-8)
Supplement: Additional file 1: — Supplementary Methods. Microsatellite markers tested for amplification in the present study and PCR protocol. (DOC 45 kb) [file 12863_2017_484_MOESM1_ESM.doc]

**Additional File 1**

**Supplementary Methods**

**Microsatellite markers tested for amplification in the present study**

| **Marker** | **Originally developed**  **for** | **Originally developed**  **by** |
| --- | --- | --- |
| Afu 54 | *A. fulvescens* | May et al., 1997 |
| Afu 68 | *A. fulvescens* | May et al., 1997 |
| Spl 101 | *S. platorynchus* | McQuown et al., 2000 |
| Spl 105 | *S. platorynchus* | McQuown et al., 2000 |
| Spl 163 | *S. platorynchus* | McQuown et al., 2000 |
| Spl 173 | *S. platorynchus* | McQuown et al., 2000 |
| Aox 45 | *A. oxyrinchus* | King et al., 2001 |
| AfuG 54 | *A. fulvescens, A. medirostris* | Welsh et al., 2003 |
| AfuG 135 | *A. fulvescens, A. medirostris* | Welsh et al., 2003 |
| Atr 109 | *A. transmontanus* | Rodzen and May, 2002 |
| Atr 114 | *A. transmontanus* | Rodzen and May, 2002 |
| Psp-12 | *P. spathula* | Heist et al., 2002 |
| Psp-18 | *P. spathula* | Heist et al., 2002 |
| Psp-20 | *P. spathula* | Heist et al., 2002 |
| Psp-21 | *P. spathula* | Heist et al., 2002 |
| Psp-26 | *P. spathula* | Heist et al., 2002 |
| Psp-28 | *P. spathula* | Heist et al., 2002 |
| Psp-29 | *P. spathula* | Heist et al., 2002 |
| Psp-32 | *P. spathula* | Heist et al., 2002 |

PCR protocol used in the present study:

PCR were performed on a volume of 25 μl, containing 1 U Taq DNA polymerase, 10 pmol of reverse primer, fluorescently labeled (VIC, NED, PET or FAM) M13R primer, 1pmol of M13R tailed forward primer, 10–50 ng DNA, 100 μM of each dNTP, 25 mM MgCl_2_, and 2.5 μl 10 x incubation buffer.

Amplifications were performed under the following conditions: one cycle at 94°C for 3 min, 94°C for 45s, 65°C for 45s, lowered annealing temperature by 1.0°C per cycle – nine cycles in total, 94°C for 45s, 52°C for 30s, 72°C for 45s, 20 cycles in total and final extension at 72°C for 10 min. The PCR products were inspected on agarose gel, then run in the ABI 3500 DNA analyzer. Genotypes were scored using GeneMapper v4.1 (Applied Biosystems, TM).

**References**

Heist EJ Nicholson EH, Sipiorski JT, Keeney DB. Microsatellite markers for the paddlefish (*Polyodon spathula*). Conserv Genet. 2002; 3: 205 – 207.

King TL, Lubinski BA, Spidle AP. Microsatellite DNA variation in Atlantic sturgeon *Acipenser oxyrinchus oxyrinchus*: and cross-species amplification in the Acipenseridae. Conserv Genet. 2001;2:103–119.

May B, Krueger CC, Kincaid HL. Genetic variation at microsatellite loci in sturgeon: primer sequence homology in Acipenser and Scaphirhynchus. Can J Fish Aquat Sci. 1997; 54:1542–1547.

McQuown EC, Sloze BL, Sheehan RJ, Rodzen J, Tranah GJ, May B. Microsatellite analysis of genetic variation in sturgeon (Acipenseridae): new primer sequences for Scaphirhynchus and Acipenser. Trans Am Fish Soc. 2000; 129:1380–1388.

Rodzen JA, May B. Inheritance of microsatellite loci in the white sturgeon (*Acipenser transmontanus*). Genome. 2002; 45:1064–1076.

Welsh AB, Blumberg M, May B. Identification of microsatellite loci in lake sturgeon, *Acipenser fulvescens*, and their variability in green sturgeon, *A. medirostris*. Molec Ecol Notes. 2003; 3:47–55.
