# Supplementary material for: Molecular cytogenetic differentiation of paralogs of Hox paralogs in duplicated and re-diploidized genome of the North American paddlefish (Polyodon spathula)
Source: BMC Genet. 2017 Mar 2;18:19. doi: 10.1186/s12863-017-0484-8 (PMC5335500; doi:10.1186/s12863-017-0484-8)
Supplement: Additional file 2: — Supplementary Results. Results of microsatellite genotyping, comparison of number of alleles, allele frequencies by locus. (PDF 83 kb) [file 12863_2017_484_MOESM2_ESM.pdf]

Additional File 2

Supplementary Results

Table S1a. Genotyping results of 24 individuals processed for microsatellite analysis.

	Lokus															
**Sample**	**Afu 68**		**Psp 12**		**Psp 18**		**Psp 20**		**Psp 21**		**Psp 28**		**Psp 29**		
PS1	158	162	234	238	192	194	220	222	164	168	266	266	210	218	226
PS2	158	170	234	234	192	194	220	222	164	168	256	268	210	218	226
PS3	162	162	234	234	184	192	220	222	164	168	256	266	210	226	
PS4	162	170	234	238	184	192	220	222	164	168	266	266	210	226	
PS5	162	162	234	234	184	192	222	224	164	168	256	266	210	226	
PS6	158	170	234	234	184	186	220	222	164	168	256	268	210	226	
PS7	158	170	234	234	186	186	220	222	164	168	256	268	210	218	226
PS8	162	162	234	234	184	186	220	222	168	168	256	266	210	218	226
PS9	158	170	234	234	186	194	220	222	164	168	256	268	210	218	226
PS10	158	170	234	234	184	186	220	222	168	168	256	268	210	218	226
PS11	170	170	234	234	184	192	220	222	164	168	256	266	210	218	226
PS12	162	182	226	226	184	192	222	222	164	168	242	266	210	226	
PS13	158	170	234	238	184	186	222	224	164	168	256	268	210	226	
PS14	158	170	234	234	184	186	220	224	164	168	256	268	210	218	226
PS15	162	170	234	238	186	194	222	224	164	168	266	266	210	226	
PS16	158	162	234	238	192	194	220	224	164	164	266	266	210	218	226
PS17	162	162	234	234	192	194	220	224	164	168	256	266	210	218	226
PS18	158	162	234	238	186	194	220	222	164	168	266	266	210	218	226
PS19	158	170	234	234	186	194	220	222	168	168	256	268	210	218	226
PS20	170	182	232	234	184	184	220	222	164	168	256	266	210	226	
PS21	170	170	234	238	186	194	220	222	168	168	256	266	210	226	
PS22	158	162	234	234	192	194	222	224	164	164	266	266	210	218	226
PS23	158	162	234	234	184	192	220	222	164	164	266	266	210	218	226
PS24	158	162	234	234	186	194	220	222	168	168	266	266	210	226	

Table S1b. Comparison of number of alleles, estimated ploidy at locus, expected heterozygosity (H_e) and observed heterozygosity (H_o) found in present study and in Heist et al. 2002.

	Locus						
	**Afu 68**	**Psp 12**	**Psp 18**	**Psp 20**	**Psp 21**	**Psp 28**	**Psp 29**
Number of alleles in present study	4	4	4	3	2	4	3
Number of alleles in Heist et al. 2002	data not available	6	6	4	7	14	4
Ploidy estimated at the locus	2n	2n	2n	2n	2n	2n	4n
Ploidy estimated at the locus in Heist et al. 2002	data not available	2n	4n	2n	2n	2n	4n
H_e present study	0.690	0.350	0.748	0.612	0.497	0.624	-
H_e Heist et al. 2002	-	0.613	-	0.562	0.721	0.889	-
H_o present study	0.750	0.333	0.917	0.958	0.667	0.667	-
H_o Heist et al. 2002	-	0.571	-	0.714	0.714	0.929	-

Table S1c. Allele frequencies by locus

Lokus											
**Afu 68**		**Psp 12**		**Psp 18**		**Psp 20**		**Psp 21**		**Psp 28**	
**Allele**	**Freq.**	**Allele**	**Freq.**	**Allele**	**Freq.**	**Allele**	**Freq.**	**Allele**	**Freq.**	**Allele**	**Freq.**
**158**	0.292	**226**	0.042	**184**	0.271	**220**	0.396	**164**	0.458	**242**	0.021
**162**	0.354	**232**	0.021	**186**	0.271	**222**	0.458	**168**	0.542	**256**	0.313
**170**	0.313	**234**	0.792	**192**	0.229	**224**	0.146			**266**	0.500
**182**	0.042	**238**	0.146	**194**	0.229					**268**	0.167
